# Development of a Buckwheat Allergy in a Child During Food Protein-Induced Enterocolitis Syndrome Due to Hen’s Egg: A Case Report

**DOI:** 10.7759/cureus.75671

**Published:** 2024-12-13

**Authors:** Yoshinori Morita

**Affiliations:** 1 Department of Pediatrics, IMS Group, IMS Memorial Hospital, Tokyo, JPN

**Keywords:** allergy to food, buckwheat, egg, food protein-induced enterocolitis syndrome, infant

## Abstract

Food protein-induced enterocolitis syndrome (FPIES) is a non-immunoglobulin E (IgE)-mediated food allergy. IgE sensitization to the causative food is often not observed, and the rate of sensitization to other common foods is not exceptionally high. This report discusses the case of a boy being followed up for FPIES due to egg yolk, who developed a buckwheat allergy during the disease. The boy was seen at the age of nine months because he vomited multiple times three hours after consuming egg yolk. Blood test results were IgE 32 IU/mL, egg white 3.78 UA/mL, and ovomucoid <0.1 UA/mL. The diagnosis was egg yolk FPIES, and a stepwise challenge test was performed; egg yolk FPIES was in remission by the age of three years. However, he developed hives after eating buckwheat when he was two years and two months old; a blood test was performed, which showed IgE 981 IU/mL and buckwheat >100 UA/mL. Initially, his condition was a non-IgE FPIES, showing that even if a patient does not have other IgE-mediated food allergy complications, it is possible to develop a new food allergy during the disease. This case demonstrates that in patients with FPIES, it is important to regularly evaluate blood tests and intake of typical causative foods for allergies.

## Introduction

Food protein-induced enterocolitis syndrome (FPIES) is a non-immunoglobulin E (IgE)-mediated food allergy that typically develops in early childhood. It is characterized by recurrent and prolonged vomiting beginning approximately one to four hours after food ingestion [1]. The foods that cause FPIES vary from country to country; however, in recent years, there has been an increase in the number of cases of FPIES caused by eggs in Japan and Turkey [2-4]. As FPIES is a non-IgE-mediated condition, IgE sensitization is often negative. However, at the time of diagnosis, 2-20% of patients show a positive reaction to FPIES-related foods, and 20-40% show a positive reaction to other general foods. Because of this, an IgE test may be considered, depending on the patient's medical history [1]. However, whether new IgE-mediated food allergies (IgE-FAs) develop during FPIES remains unclear. This report describes the case of a child who was being followed up for egg yolk-induced FPIES and subsequently developed a buckwheat allergy.

## Case presentation

A male child at nine months of age vomited four hours after eating commercially-produced egg-containing rice-based weaning food. At 10 months of age, he vomited five hours after consuming one-quarter of a boiled egg. At 11 months of age, he vomited and felt faint three hours after ingesting 0.5 g of egg yolk. In all episodes, he did not develop hives or experience coughing. Because of recurrent reactions to eggs, the patient was referred to our department from a local clinic. The patient had a history of mild eczema, and a previous doctor had prescribed topical steroids, and the symptoms were well controlled. No other physical findings were observed. The family history revealed that the father had allergic rhinitis. Blood test results showed a total IgE of 32 IU/mL, egg white-specific IgE of 3.78 UA/mL, and ovomucoid-specific IgE <0.1 UA/mL. Based on these test results and symptoms, the child was diagnosed with egg yolk-induced FPIES. Initially, a total egg-elimination diet was recommended, and the child's progress was monitored. Because the patient was sensitized to egg whites, an oral food challenge (OFC) for both egg whites and egg yolks was conducted at the hospital, with gradual increases in the amount and continued intake (Table 1).

**Table 1:** Amount of food consumed in the oral food challenge, which passed

	Age: 1 y 9 m	Age: 2 y	Age: 2 y 4 m	Age: 2 y 8 m	Age: 3 y
Egg yolk (g)	-	0.1	0.5	2.5	-
Egg white (g)	5	-	-	-	-
Whole egg (g)	-	-	-	-	20

The amounts tolerated during the OFC could be continued at home without any problems. However, when the patient then consumed buckwheat at two years and two months, urticaria developed throughout the body. Blood tests showed a total IgE level of 981 IU/mL and a buckwheat-specific IgE level >100 UA/mL. In addition to FPIES, the only allergic disease observed was mild eczema, which was well-controlled with topical steroids. At the age of three, it was determined that the egg yolk-induced FPIES was in remission, and it was confirmed that he could consume cooked whole eggs without complications. However, as shown in Table 2, the patient's total and specific IgE levels indicated that buckwheat-specific IgE levels remained elevated, presenting ongoing treatment challenges.

**Table 2:** Results of total and specific IgE levels during the course of the disease -: This specific IgE measurement was not performed at the corresponding time point. IgE: Immunoglobulin E

	Age: 11 m	Age: 2 y 2 m	Age: 3 y 6 m
IgE (IU/mL)	32	981	1463
Specific IgE (UA/mL)			
Egg white	3.78	-	1.66
Egg yolk	-	-	0.84
Ovomucoid	< 0.35	-	< 0.35
Buckwheat	-	> 100	> 100
Peanuts	< 0.35	-	0.43
Walnuts	< 0.35	< 0.35	< 0.35
Cashew nuts	-	< 0.35	< 0.35
Almond	-	-	< 0.35
Hazelnut	-	-	< 0.35

## Discussion

Examining the course of FPIES in this case, the findings indicate that even if the initial presentation of FPIES is non-IgE-mediated and other weaning foods are introduced without problems, developing a new IgE-FA is possible. Second, even if the initial total IgE level is low and a child with FPIES exhibits poor sensitization, the allergic background can change significantly over time. This indicates that even if the course of FPIES is favorable, it is essential to monitor for potential changes. Whether new IgE-FAs develop during FPIES remains unclear. As FPIES is a non-IgE-mediated condition, in most cases, there is no evidence of allergic sensitization, even when it comes to causative food. At the time of diagnosis, 2-20% of patients may show a positive reaction to FPIES-related foods, and 20-40% may show a positive reaction to other common foods [1]. Although it has been suggested that food sensitization in FPIES may be associated with a transition to IgE-FA or difficulty in achieving remission [5], other reports indicate that food sensitization does not necessarily affect prognosis [6,7].

The prognosis of FPIES, whether or not there is sensitization, differs depending on the causative agent. In the case of FPIES caused by egg yolk, more than half of patients tolerate it between the ages of 20 months and three years [7-9]. In this case, the patient passed egg white in the oral food challenge (OFC) and consumed it without any issues. He achieved tolerance to egg yolk by three years, which is typical for egg yolk-induced FPIES. In the current case, the child initially had a low total IgE level and showed no sensitization to peanuts or tree nuts, common allergens in Japan at young ages. The observations indicate that IgE-FA can develop in response to new foods, even in FPIES with a good prognosis. 

In cases of non-IgE-mediated FPIES, determining the extent to which allergic status should be evaluated during the disease can be challenging. For patients with other atopic conditions, such as atopic dermatitis (AD), wheezing, or IgE-FA, testing for FPIES-inducing substances (such as eggs, peanuts, tree nuts, sesame, fish, and crustaceans) is recommended [10]. In this case, only mild eczema was observed. Based on the total IgE level and other factors, it was initially thought that there were no issues with other foods. However, the total IgE level significantly increased during the course of the disease, and the patient developed a typical atopic status. 

In a nationwide survey of food allergies in Japan, buckwheat was ranked 11th as a causative food, accounting for 1.1% of all immediate-type food allergies [11]. The routes of sensitization to buckwheat include occupational exposure and use of buckwheat pillows [12]. Although the child was highly sensitized to buckwheat, the family's occupation was unrelated to buckwheat, and none of his households used buckwheat pillows. There was a buckwheat flour mill near the family home, but the extent of its impact on the home environment remains to be determined. Moreover, AD control was consistently stable throughout the disease course, requiring minimal topical steroids, and there was no history of asthma. The patient tolerated other foods, such as peanuts, tree nuts, and rice, which are common allergens reported to be cross-reactive with buckwheat [12], without adverse reactions. However, poppy seeds were not evaluated due to the limited opportunities for consumption in Japan. The clinical course suggested the possibility of sensitization via the airway or skin rather than cross-reactivity with other foods; however, the reason why this patient was specifically sensitized to buckwheat remains unclear. In this case, the patient developed a new allergy to buckwheat even though his FPIES and coexisting AD were well controlled. This case suggests that even in cases of FPIES, it is essential to monitor the consumption of other foods and regularly perform blood tests, as is done for IgE-FA.

## Conclusions

Even if the initial diagnosis was non-IgE-mediated FPIES, new IgE-FAs may develop over time. Our case findings suggest that careful attention should be paid regardless of the FPIES course. Allergic status may change significantly; thus, caution is required when IgE levels fluctuate greatly. Regular assessments of the intake of common causative foods and allergic status are critical, as in the management of IgE-FAs.
